# Potential Albumin-Based Antioxidant Nanoformulations for Ocular Protection against Oxidative Stress

**DOI:** 10.3390/pharmaceutics11070297

**Published:** 2019-06-26

**Authors:** Daseul Kim, Pooja Maharjan, Minki Jin, Taehoon Park, Anjila Maharjan, Reeju Amatya, JaeWook Yang, Kyoung Ah Min, Meong Cheol Shin

**Affiliations:** 1 College of Pharmacy and Inje Institute of Pharmaceutical Sciences and Research, Inje University, 197 Injero, Gimhae, Gyeongnam 50834, Korea; 2 College of Pharmacy and Research Institute of Pharmaceutical Sciences, Gyeongsang National University, 501 Jinju Daero, Jinju, Gyeongnam 52828, Korea; 3 Department of Ophthalmology, College of Medicine, Inje University, 75 Bokjiro, Busanjin-gu, Busan 47392, Korea; 4 T2B Infrastructure Center for Ocular Diseases, Inje University Busan Paik Hospital, 75 Bokjiro, Busanjin-gu, Busan 47392, Korea

**Keywords:** antioxidant, bovine serum albumin, ophthalmic formulation, drug transport, cornea, retinal pigmented epithelium

## Abstract

Amongst various drug administration methods, ophthalmic drug delivery has been a useful way for the treatment of eye-related diseases. However, therapeutic efficacy of ocular therapy for anterior or posterior eye segments through topical administration is considerably challenged by the number of anatomical and physiological barriers in the eyes affecting ocular bioavailability. In this respect, advanced biocompatible nanoformulations make it possible to improve drug delivery to the target sites and enhance ocular bioavailability of ophthalmic medicines. Various ocular diseases have been reported to be related to oxidative stresses in tissues, and polyphenolic compounds have been known for their antioxidant activities in various tissues, including the eyes. Despite drug efficacy, poor water solubility and intrinsic color of the compounds limit the drug’s inclusion into the development of ocular medicine. In the present study, we investigated the antioxidant protectant efficacy of rosmarinic or ursolic acid in the retinal epithelial cells, as compared to those of curcumin, by forming nanospheres with bovine serum albumin. Our results demonstrate that antioxidant-containing nanoformulations provide a significantly higher drug solubility and decreased ROS (reactive oxygen species) production in the retinal epithelial cells. Finally, we also found that albumin-based nanoformulations could improve bioavailability and increase antioxidant activity of rosmarinic or ursolic acid in the retina to be applied as efficient ocular protectant.

## 1. Introduction

Because of the unique pharmacodynamics and pharmacokinetic properties of the eyes, characterized by anatomical structures of anterior and posterior segments, and tear dilution, ophthalmic delivery system faces challenges in administering drugs [1]. Pharmacodynamics and pharmacokinetic properties of the eyes create therapeutic challenges and result in poor ocular bioavailability [2]. Therefore, there is an urgent need for advances in a nanoengineering drug delivery system for an effective delivery of drugs to target sites along with improved ocular bioavailability. In this respect, different nanoengineered strategies have been investigated to improve the drug delivery system via the ocular route for the treatment of various ocular diseases [2]. Various biocompatible drug carriers have been incorporated to ensure optimal ocular delivery formulations so as to increase effective drug concentrations in anterior or posterior eye tissues.

Oxidative stress is one of the predisposing factors of various ocular diseases such as age-related macular degeneration, corneal neovascularization, diabetic retinopathy, or dry eye syndrome [3,4,5]. Phenolic compounds are known as potential antioxidants that can control excessive production of ROS, which eventually leads to oxidative stress [6]. Among different types of phenolic compounds, curcumin, a yellow colored polyphenol compound, has recently gained considerable scholarly attention because of its beneficial properties, such as anti-inflammatory activity. Therefore, numerous studies have investigated its antioxidant properties, as curcumin prevents tissue damage by lipid peroxidation by inhibiting free radicals or reactive oxygen species (ROS) production [6]. ROS is a natural byproduct of cellular metabolism produced by mitochondria during cell respiration. Under physiological conditions, the balance between ROS generation and clearance is managed by various antioxidant defense mechanisms, such as superoxide dismutase (SOD) and glutathione peroxidase (GPX), the key antioxidant enzymes in balancing ROS production [7]. Basically, SOD1 dissociates the superoxide to oxygen and hydrogen peroxide, and GPX1 is known as a key enzyme for the defense of cells against oxidative stresses by eliminating H_2_O_2_ [8]. However, the therapeutic efficacy of curcumin is limited because of its poor aqueous solubility (approximately 10–20 µg/mL), which eventually affects ocular bioavailability. Furthermore, there are limitations of curcumin, which is a pigmented compound, in terms of using it as a drug applied to the ocular tissues. In this aspect, rosmarinic acid (RosA) or ursolic acid (UrsA), both of which are noncolored, transparent antioxidant compounds, can substitute curcumin for treatment of ocular diseases. At the same time, these compounds also show lipophilic properties, resulting in their poor water solubility, which limits their extraordinary therapeutic properties [9,10]. However, the strategic design of a nanoengineered formulation containing these candidates would increase their solubility and, ultimately, enhance their ocular bioavailability [11].

Several approaches have attempted to circumvent the limitations of curcumin, RosA, or UrsA to increase the solubility of these compounds so that the pharmaceutical functions of such poorly soluble compounds can be fully used. For instance, formulations with nanospheres, nanocapsules, or a nanoparticle matrix have been prepared to yield an applicable drug delivery system despite the challenges in normal eye physiology [12,13]. Amongst various carriers, protein-based nanoparticles offer interesting merits, such as being nontoxic, nonimmunogenic, and having a better stability during prolonged storage [14,15]. In addition to these aspects, they have attracted researchers because they are easy to scale up in the manufacturing process as compared to other drug delivery systems [16]. Among different types of protein, albumin is considered to be an attractive macromolecular carrier because of its multiple advantages such as, for instance, its nonimmunogenic, nontoxic, biocompatible, and biodegradable properties. Therefore, albumin can be considered as a potent drug carrier to increase the solubility of poorly soluble compounds, like curcumin, RosA, or UrsA, and to improve drug transport from the drug depot to the target sites. Moreover, albumin-based nanoparticles are considered to be an attractive strategy, since different drug binding sites are present in the albumin molecule [17], and a significant amount of the drug can be incorporated into the particle matrix. In addition, the primary structure of albumin has a high content of charged amino acids, such as lysine, thereby allowing electrostatic adsorption of both positively and negatively charged molecules without any catalysts. Among various types of albumin—ovalbumin, bovine serum albumin (BSA), and human serum albumin—bovine serum albumin has wide medicinal applications, particularly in the field of drug delivery, owing to its abundance, low cost, and ease of purification. In addition, lower ligand-binding properties make it attractive in the pharmaceutical industry [16]. There are reports showing that the chemical stability of curcumin in physiological conditions has been improved after binding with BSA because it can provide excellent steric and electrostatic stabilization of nanoparticles by virtue of the presence of adsorbed protein coronas on the surface of nanoparticles [18,19]. Thus, herein, we prepared nanoformulations that contained curcumin, RosA, or UrsA using albumin as a drug carrier, and they were further characterized by evaluating various physicochemical characteristics.

To this end, we assessed differential scanning calorimetry (DSC), Fourier transform infrared (FT-IR) spectrometer, and microscopic imaging to characterize the formulation properties of BSA particles containing curcumin, RosA, or UrsA. Additionally, a WST-1 assay was performed to evaluate the cytotoxicity of drug formulations in different concentrations with adult retinal pigmented epithelial (ARPE) cells. Finally, the optimal concentrations of each drug formulation were used to evaluate antioxidative protection properties in both in vitro and ex vivo experiments.

## 2. Materials and Methods

### 2.1. Materials

Bovine serum albumin (heat shock fraction, ≥98%), 25% glutaraldehyde solution, curcumin (≥65% by HPLC), rosmarinic acid (96%), ursolic acid (≥90%), acetone, xylene, and ethanol were purchased from Sigma Aldrich Co. (St. Louis, MO, USA). Dulbecco’s phosphate buffered saline (DPBS) and basal Dulbecco’s Modified Eagle Medium/F12 (1:1) were purchased from Invitrogen (Carlsbad, CA, USA). Media supplements such as fetal bovine serum (FBS), penicillin–streptomycin, and the subculture solution (0.25% Trypsin-EDTA) were obtained from Invitrogen. Human adult retinal pigmented epithelial (ARPE) cells from ATCC (Manassas, VA, USA) and culture well plates from Corning Life Sciences (Lowell, MA, USA) were used for cell studies. A QuantiTect Reverse Transcription Kit (Qiagen, Hilden, Germany and TOPreal ™ qPCR reagents (Enzynomics, Daejeon, Republic of Korea) were utilized for qRT-PCR experiments. Hematoxylin solution (YD Diagnostics, Yongin, Republic of Korea) and Eosin Y (Muto Pure Chemicals Co., Tokyo, Japan) were used for histological assays.

### 2.2. Preparation of Albumin-Based Drug Formulations

Albumin-based nanoformulations were prepared using the previously described desolvation method [19] with some modifications. Briefly, bovine serum albumin (BSA) was dissolved in HPLC-grade water to reach the concentration of 10 mg/mL; then, 12 mL of ethanol solution containing 8 mg of curcumin was added dropwise via a syringe at the rate of 1 mL/min into 2 mL of BSA solution stirred at 500 rpm in room temperature. For sufficient desolvation, the BSA solution with curcumin was agitated for 1 h with a cover followed by the addition of 100 μL of 0.5% glutaraldehyde solution for cross-linking. After stirring overnight in the dark, the organic solvent was evaporated using a rotary vacuum evaporator (N-1110, EYELA, Tokyo, Japan) at 25 °C. After complete evaporation of ethanol, HPLC-grade water was added to the original volume and re-dispersed. Finally, the formed nanoparticles were lyophilized to obtain a powder by using a freeze dryer at −80 °C for at least 48 h. RosA/BSA and UrsA/BSA nanoparticles were prepared using the aforementioned procedure.

### 2.3. Drug Encapsulation Efficiency

To evaluate the efficiency of drug encapsulation, the prepared nanosuspension was first centrifuged at 16,500 g for 25 min at 4 °C [20]. In the next step, free curcumin present in the supernatant was diluted with methanol, vortexed to ensure that the drug was sufficiently dissolved, and finally quantified using HPLC (Waters Co., Milford MA, USA with a UV detector. The curcumin standards of different concentrations (0–100 µg/mL) diluted with methanol were quantified to make a standard curve under the same conditions to calculate the exact drug concentration. For the HPLC analysis, the solvent mixture composed of 0.1% phosphoric acid, acetonitrile, and methanol (1:1:8) was used with the isocratic mode [21]. The absorbance of curcumin was detected at a wavelength of 425 nm with a 0.8 mL/min flow rate. Similarly, RosA or UrsA were also extracted by the same method as the one used for curcumin, whereas there were slight changes in the detection: RosA and UrsA absorbances were detected at the wavelengths of 280 and 210 nm, respectively, with a flow rate of 1.0 mL/min [22,23]. The sample solution (20 μL) was injected to HPLC and analyzed until 10 min. Finally, Equation (1) was used to determine the encapsulation efficiency (EE).

(1) $$\mathrm{EE}\;\left(\%\right)=\;\frac{(\mathrm{total}\;\mathrm{drug}\;\mathrm{added}\;–\;\mathrm{free}\;\mathrm{non}-\mathrm{entrapped}\;\mathrm{drug})\;}{\mathrm{the}\;\mathrm{total}\;\mathrm{drug}\;\mathrm{added}}\times 100$$

### 2.4. Measurement of Particle Properties by Dynamic Light Scattering (DLS) and Transmission Electron Microscopic Imaging (TEM)

Measurements of nanoparticle sizes and zeta potential values were performed using NanoBrook 90Plus instruments (Brookhaven Co., New York, USA). To measure zeta potentials, a BI-ZEL electrode assembly for aqueous systems was used. For measurement, each formulation sample before and after freeze-drying was diluted to the appropriate concentration with water. The results of the size and zeta potential were expressed as mean ± standard deviation of the three measurements. In addition, transmission electron microscopic imaging (TEM) was performed to observe the morphologies of the drug particles using JEM 2100F microscopy (JEOL Ltd., Tokyo, Japan) at 160 kV. The appropriately diluted specimen was loaded on cooper TEM grids (300-mesh carbon-coated), allowed to dry, and followed by staining with osmium tetroxide.

### 2.5. Physicochemical Properties by DSC, X-ray Diffractogram (XRD), and FT-IR

A TA Instruments Q20 Differential Scanning Calorimeter (New Castle, DE, USA) was used for differential scanning calorimetry (DSC) analyses of pure curcumin, RosA, UrsA, BSA, drug formulations (Curcumin/BSA, RosA/BSA, and UrsA/BSA), and physical mixtures (PMs) of each drug with BSA. The instrument was calibrated using indium, and sample scanning was conducted under nitrogen gas at 20 mL/min. A DSC isotherm curve was generated for specimens in an aluminum pan with a lid at a temperature range of 40–300 °C (with a heat rate of 10 °C/min).

Next, in order to assess any crystalline properties of the formula, an X-ray diffractogram (XRD) was also measured for pure curcumin, RosA, UrsA, BSA, drug formulations (C/BSA, RosA/BSA, and UrsA/BSA), and physical mixtures (PMs) of drug and BSA. An Ultima IV diffractometer (Rigaku, Japan) was used with a voltage of 45 kV and a current of 30 mA for XRD patterns. The diffraction angle (2θ) of the sample was scanned between 0° and 90°. Furthermore, chemical component analysis results for each type of drug formula were obtained with an FT-IR 6300 spectrometer (JASCO, Tokyo, Japan). Potassium bromide pellets containing a single compound, physical mixtures, and formulations were used. Data were collected at a wavelength of 4000–400 cm^−1^ at ambient temperature.

### 2.6. Drug Release Study

A dialysis bag with a membrane (molecular weight cut-off; 8–10 kDa) was utilized for drug release studies (Spectrum Laboratories, Inc., Rancho Dominguez, CA, USA) [24,25]. The sink condition was assumed to be reached by agitating the vial with the dialysis bag of drug solution. Inside the dialysis bag, 1 mL of drug solution (1 mg drug powder in 1 mL of PBS with 0.5% SLS) was included, while the solution surrounding the dialysis bag in the beaker contained 0.5% sodium lauryl sulfate (SLS) in 6 mL of PBS (pH 7.4). During the experiment, the beaker with the dialysis bag (drug solution inside the bag) was installed in a shaking water bath at 37 °C. At each time point (from 5 min until 72 h), the sample solution (200 µL) was taken from the beaker solution, and blank PBS (200 µL) with 0.5% SLS was put into the beaker. The sample at each time was filtered by a syringe filter and injected to the HPLC. Samples from the drug release study were analyzed by the established HPLC method as mentioned above. The measurements were repeated thrice.

### 2.7. Cell Culture

ARPE cells (American Type Culture Collection (ATCC), Manassas, VA, USA) were cultivated in DMEM/F12 (1:1) growth media containing 10% FBS and 100 U/mL penicillin–streptomycin. The cells were expanded with the media in flasks 75 cm^2^ at 37 °C in a 5% CO_2_ incubator. The cells were treated with Trypsin-EDTA for subculture when the cells in the flasks reached 80% confluence. The culture medium was changed regularly (every second day) for maintaining cells in the flask.

### 2.8. Cytotoxicity Test

For the assessment of cytotoxicity of prepared drug formulations, ARPE cells at a density of 1.7 × 10^5^ cells/mL were seeded in 96-well plates. After overnight incubation at 37 °C in a 5% CO_2_ incubator, the medium was removed from each well, and each well was carefully washed with DPBS. Then, the cells were incubated with drug formulations (C/BSA, RosA/BSA, and UrsA/BSA), and then cytotoxicity was evaluated by water soluble tetrazolium salt (WST)-1 reagents. Cells without treatment were used as the negative control group. For the test group, 110 μL of formulations at various drug concentrations (0, 5, 10, 30, 50, 100, or 200 μM) were added to the well plate (*n* = 3). After incubation with the formulation for 18 or 24 h and then removal of the drug solution, 100 μL of fresh medium were added to the well, followed by addition of 10 μL of cell survival detection reagent, WST-1, and then incubation at 37 °C in a 5% CO_2_ atmosphere for 1 h. Finally, the absorbance values were measured under a wavelength of 440 nm.

### 2.9. Detection of Cellular Reactive Oxygen Species (ROS) Production

Cellular ROS production was detected using DCFDA/H2DCFDA (Cellular ROS Assay Kit, Abcam^®^, Cambridge, UK) according to the manufacturer’s protocol. ARPE cells were seeded into 96-well plates at 1 × 10^5^ cells/mL. The drug formulations C/BSA, RosA/BSA, and UrsA/BSA were diluted with media to achieve 30, 50, and 100 μM concentrations of the drug. After incubating the cells for 24 h, media were removed from all wells and carefully washed with DPBS. In the negative control group, 110 μL of media was added; in the drug-treatment group, the cells were treated with 110 μL of drug solution and further incubated for 18 h. After completion of the incubation period, all solutions were removed from each well and washed with DPBS. Then, 100 μL of ROS reagent was added (20 mM stock diluted with 1 × DCFDA buffer to 25 μM) to all wells followed by further incubation for 45 min. For ROS measurements, fluorescence was measured with a cell imaging multimodal reader (CYTATION 5, BioTek Instruments, VT, USA) with excitation at 490 nm and emission at 525 nm. Cell-permeable fluorescent dye DCFDA (2′,7′-dichlorofluorescein diacetate) was deacetylated as a nonfluorescent compound by cellular esterase, which later oxidized to 2′,7′-dichlorofluorene (DCF) and is highly fluorescent by ROS, to measure hydroxyl, peroxyl, and other ROS activities in cells.

### 2.10. Assessment of Antioxidant Gene Levels by qRT-PCR

In order to detect cellular gene expression after oxidative stress and drug therapy (C/BSA, RosA/BSA, and UrsA/BSA), a quantitative real time polymerase chain reaction (qRT-PCR) was used where, initially, ARPE cells were seeded at 3.9 × 10^5^ cells/mL in 6-well plates. After confirmation of cell confluency, cells were treated with the drug formulations (30 or 50 μM) and incubated at 37 °C under 5% CO_2_ for 18 h. Next, the cells were incubated with 30 μg/mL of H_2_O_2_ as the oxidative stress agent for 4 h. Finally, total RNA was extracted with RNeasy Mini Kit^®^ (Qiagen, Valencia, Calif.), quantified with a spectrophotometer (NanoDrop 2000; Thermo Scientific, Wilmington, DE), and stored at −80 °C prior to use. Then, a Reverse Transcription Kit^®^ (QuantiTect, Hilden, Germany) was used to obtain complementary DNA from the isolated RNA. A qRT-PCR assay based on SYBR Green was performed as per the protocol provided by TOPreal ™ qPCR 2× PreMIX kit (Enzynomics, Daejeon, Korea), after which RT-PCR was performed with the following procedure: initial denaturation at 95 °C for 10 min, denaturation at 95 °C for 10 s, annealing at 55 °C for 15 s, and elongation at 72 °C for 15 s (40 cycles in total). The sets of individual gene primers for the PCR of the beta-actin were CCA-ACC-GCG-AGA-AGA-TGA (forward) and CCA-GAG-GCG-TAC-AGG-GAT-AG (reverse). The primer sets for the superoxide dismutase1 (SOD1) included TG-ACA-AAG-ATG-GTG-TGG-CCG-AT (forward) and CA-AAC-GAC-TTC-CAG-CGT-TTC-CT (reverse). The sets for the glutathione peroxidase 1 (*GPX1*) were CAA-CCA-GTT-TGG-GCA-TCA-G (forward) and GTT-CAC-CTC-GCA-CTT-CTC-G (reverse). Specification of amplified DNA was analyzed with the melting curve using the 2-ΔΔCt method [26].

### 2.11. Immunofluorescent Microscopic Imaging

ARPE cells were seeded in Lab-Tek 8-chamber slides with a density of 1.1 × 10^6^ cells/cm^2^. After treating the cells with the drug formulation and then creating oxidative stress by the addition of 30 μg/mL H_2_O_2_, as explained in qRT-PCR, the cells were fixed with freshly prepared 4% formalin for 10 min at 4 °C. Then, the cells were incubated with 0.2% Triton X-100 in PBS at room temperature for 20 min for permeabilization, followed by incubation with the primary antibody against human superoxide dismutase 1 (SOD1) or glutathione peroxidase 1 (GPX1). After overnight incubation at 4 °C, Alexa-Fluor 488 conjugated secondary antibody and propidium iodide (PI) for nuclear staining were then applied. Finally, immune-stained cells were observed with a Zeiss Axio observer Z1 with Axiocam MRm (ZEISS, Thornwood, NY).

### 2.12. Protective Effects against Oxidative Stress

The animal tissue experiment was performed following the guidelines of Inje University Busan Paik Hospital, approved by the Institutional Animal Care and Use Committee (No. IJUBPH_2018-011-06, August 2018). New Zealand white rabbits weighing 1.5–2.0 kg from Samtako (Republic of Korea) were used for the drug efficacy studies. Animal corneas and retinas were removed from the rabbit eyes and washed with PBS (pH 7.4). The setup with glass diffusion cells was used to evaluate protectant properties of the drug formulations (C/BSA, RosA/BSA, or UrsA/BSA) against oxidative stress. The corneas or retinas were carefully put between side-by-side type diffusion cells set at a temperature of 34 °C. In the case of the positive control, the tissue was faced with 30 µg/mL of H_2_O_2_ (5 mL) in the donor cell, while there was 5.5 mL of blank Hank’s balanced salt solution (HBSS; pH 7.4) in the receiver cell. The drug formulation solution was diluted to 50 μM using HBSS (pH 7.4). The corneas or retinas in the drug treatment group were incubated with drug/BSA formulation (5 mL; Cur-BSA, RA-BSA, or UA-BSA) in the donor cells for 18 h, then they were treated with 30 μg/mL of H_2_O_2_ solution (5 mL) for 4 h. After completion of the animal tissue experiment, tissues were fixed with 4% formalin solution (Sigma Aldrich, USA) and stained by hematoxylin and eosin (H&E) reagents for histological observation under the microscopy.

### 2.13. Data Analysis

GraphPad Prism software (ver. 5.03; LaJolla, USA) was used for data analysis. One-way ANOVA tests were performed with Tukey’s multiple comparison tests as a post hoc analysis at a significance level of 0.05.

## 3. Results and Discussion

### 3.1. Physical Characteristics and Stability of Drug/Bovine Serum Albumin (BSA) Formulations

**Figure 1:**
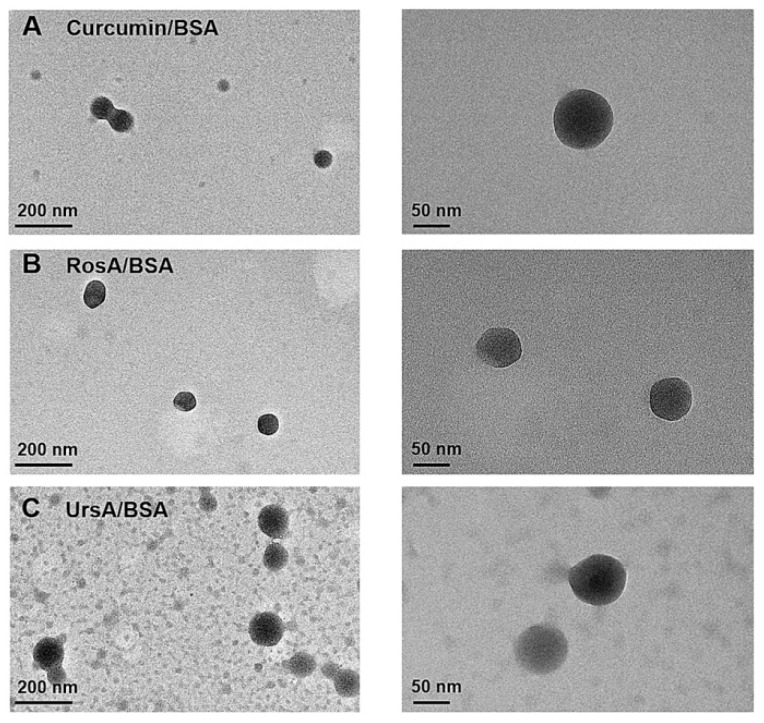
Transmission electron microscopic imaging (TEM) of the drug/BSA nanoparticles. (**A**) curcumin/BSA, (**B**) RosA/BSA, and (**C**) UrsA/BSA. The scale bars are displayed on each image.

In this study, natural protein bovine serum albumin (BSA) was used to prepare stable drug formulations containing the lipophilic drug molecules such as curcumin, rosmarinic acid (RosA), or ursolic acid (UrsA). Particle sizes and zeta potential values were measured to confirm the physical stability of the drug/BSA formulation in solution and powder form during the storage period, as shown in Table 1. All drug solution types were diluted 5 times in water, while 3 mg of drug powder was dispersed in 3 mL of water for dynamic light scattering (DLS) measurements. Zeta potential values of all drug formulations were negatively charged in the aqueous dispersion. During storage at 4 °C (for 2 months), the zeta potentials of each drug-type formulation in both solution and freeze-dried formulations varied only slightly from those at initial preparation. After 2 months of storage, the average particle sizes of C/BSA and UrsA/BSA in a solution state were approximately 1.7-fold higher than that in the initial state, whereas the particle sizes in the freeze-dried powder changed little. DLS size measurements based on number distribution and transmission electron microscopy (TEM) imaging (Figure 1) confirmed the drug preparations based on BSA had nanosize ranges (~100 nm) in spherical shapes. TEM images exhibited spherical, amorphous particles of curcumin, RosA, or UrsA in albumin. As shown in the images (Figure 1) and polydispersity (PDI) values of DLS (Table 1), BSA formulations of curcumin, RosA, or UrsA were prepared in consistent sizes and shapes. PDI values of the hydrodynamic sizes confirmed the uniformity in the final preparations of each formulation. During two months of storage in solution or freeze-dried powder, albumin formulations of these drugs showed drug loading efficiencies similar to the initial values (%), maintaining >90% encapsulation efficiency (data not shown). As other studies suggest, the albumin particles showed better storage stability of these lipophilic drug molecules than other hydrophilic carriers (for example, cyclodextrins) [16,27].

### 3.2. Drug Crystalline Properties of Drug/BSA Formulations

**Figure 2:**
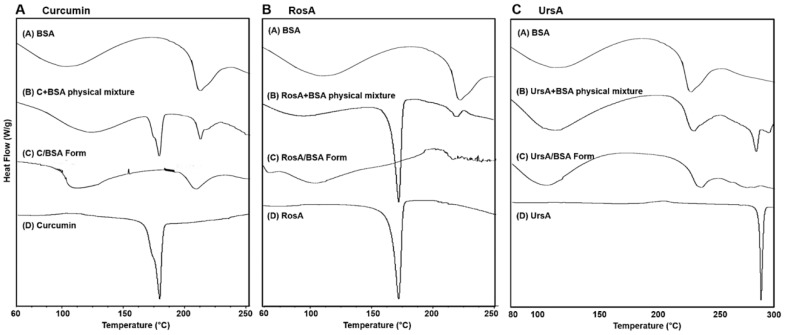
Differential scanning calorimetry (DSC) data of BSA-based formulations with pure drug compounds and physical mixtures of drug and BSA. The results show the DSC peak patterns including (**A**) curcumin (C), (**B**) rosmarinic acid (RosA), and (**C**) ursolic acid (UrsA).

Drug/BSA formulations were further characterized for their physicochemical properties by DSC (differential scanning calorimetry) and XRD (X-ray diffractogram). As the drug molecules were encapsulated in the albumin, the endothermic peaks of the pure compounds disappeared, and the DSC peak patterns of the drug/BSA formulations looked similar to those of the pure BSA peaks (Figure 2A vs. 2C for curcumin, RosA, and UrsA). The prominent peaks of curcumin (176 °C), RosA (171 °C), and UrsA (286 °C) disappeared, showing amorphous characteristics in the albumin formulations. The endothermic peaks of the BSA at 101 and 222 °C shifted slightly in temperature in the cases of the drug/BSA formulations, suggesting the interaction between the drug molecules and albumin changed the molecular confirmation in the protein. Generally, it is known that the endothermic peaks of the compound could disappear or shift by inclusion of the drug molecule into the vehicles [28]. The phenomenon of changing melting points reflects the crystal lattice structures of molecules [29]. In physical mixtures (Figure 2B for drug-types), there were peaks of BSA and pure drug compounds, with shifted peaks of BSA, which meant there were molecular interactions between the drugs and proteins.

**Figure 3:**
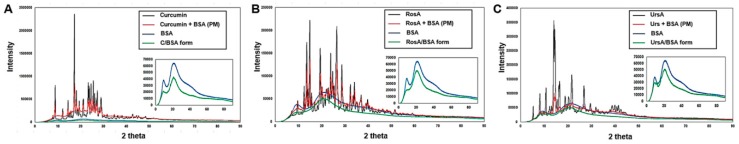
X-ray diffraction results with BSA nanoparticles of (**A**) curcumin, (**B**) RosA, and (**C**) UrsA, along with the data on pure compounds (drug or BSA powder) or physical mixtures.

XRD results also showed that the physical state of the drug compounds changed into the amorphous form, without crystalline peaks, when encapsulated in the albumin nanoparticles (Figure 3). The thermograms apparently exhibited that the crystalline peaks of pure compounds disappeared in drug/BSA formulations. Similar peaks to those of pure BSA were shown in the formulations. In the physical mixture (PM) of each drug-type with BSA, the characteristic peaks of curcumin, RosA, and UrsA had reduced intensities, as compared with those of pure curcumin. Therefore, these DSC and XRD measurements also confirmed that the albumin particles were properly prepared to include those lipophilic drugs.

### 3.3. Fourier-Transform Infrared Spectrum Results of Drug/BSA Formulations

**Figure 4:**
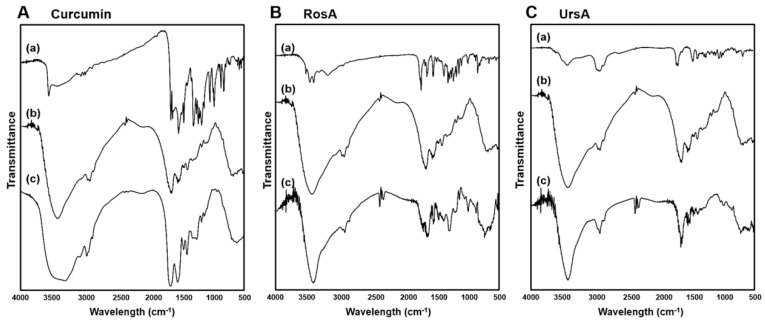
FT-IR spectrum with the BSA nanoparticles of (**A**) curcumin, (**B**) RosA, and (**C**) UrsA along with the data on pure compounds (drug or BSA powder) or physical mixtures.

FT-IR spectrum analyses were performed to confirm the chemical component of each drug formulation with BSA. Characteristic peaks of curcumin ranged between 1700–600 cm^−1^ bands for (a) pure curcumin (see Figure 4A). Strong peaks of curcumin exhibited strong peaks at 1597 and 1504 cm^−1^ associated with aromatic C=C and C=O vibrations in its benzene ring. Additionally, olefinic C–H bending vibrations were found at 1426 cm^−1^, aromatic C–O stretching vibrations at 1270 cm^−1^, and C–O–C stretching vibrations at 960 cm^−1^. The characteristic adsorption bands of (b) BSA at 1625 and 1509 cm^−1^ were attributed to the vibration adsorption of amide I (–N–H_2_) due to C=O stretching and amide II (N–H bending) coupled with C–N stretching, respectively. These were the most prominent vibrational bands in the albumin backbone to form the secondary structure of the protein. In the case of (c) curcumin/BSA formulations, distinct peaks of pure curcumin were hidden by the BSA peaks, demonstrating the encapsulation of curcumin in the BSA protein (Figure 4**A**). By the chemical interaction between curcumin and albumin, amide I peak and II peak positions shifted to 1629 and 1522 cm^−1^, respectively. This suggests that the interaction between drug and C=O and C–N groups in BSA might have changed the secondary structure of BSA [30].

In can be seen from Figure 4B that the IR spectrum of RosA showed a broad adsorption band at 3117 cm^−1^ (O–H of carboxylic acid) in addition to other bands at 1705 (C=O of carboxylic acid), 1606 (C=O of conjugated with double bond), 1511, and 1415 cm^−1^ (stretching of aromatic ring). Comparison of IR spectra between (b) pure BSA and (c) RosA/BSA formulation confirmed there was a chemical interaction between RosA and albumin because there was a change in intensity and peak shift of each amide I and II bands.

For the (a) pure ursolic acid (Figure 4C), characteristic peaks were observed at 3403 (O–H stretching vibrations), 2923, 2898 (C–H stretching vibrations peaks), 1683(stretching vibration of carbonyl group), and 1442–1035 cm^−1^ (characteristic peaks of ursane triterpenoids). The peaks of (c) UrsA/BSA formulation demonstrated the chemical interaction between UrsA and albumin, with shifted positions of amide I and II peaks to 1638 and 1508 cm^−1^, respectively. Based on the IR spectrum results, these drug compounds were found to interact with polypeptide chains in BSA, changing the secondary structure of BSA by a complex formation [31]. These physicochemical results support that the preparations of albumin-based nanoparticles possessed high complexation efficiency to include lipophilic compounds.

### 3.4. Drug Release Profiles of Drug/BSA Formulations

**Figure 5:**
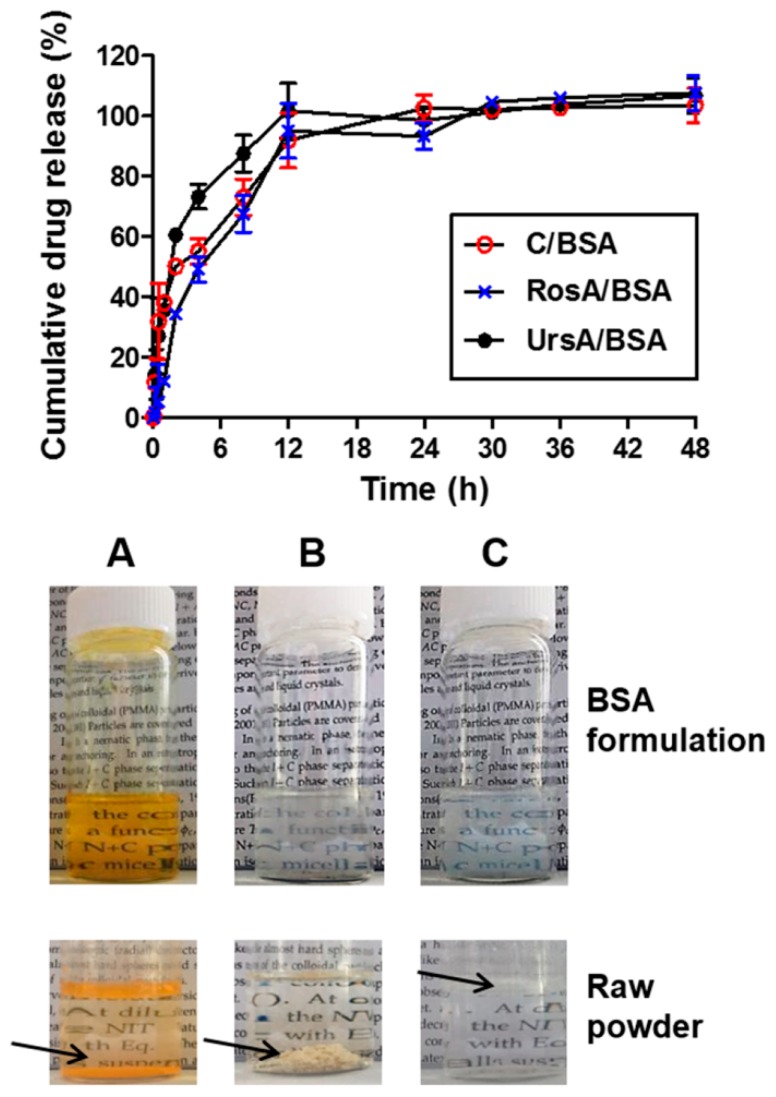
Drug release profiles of the BSA nanoparticles (C/BSA, RosA/BSA, and UrsA/BSA). Pictures of vials show the drug/BSA formulations with (**A**) curcumin, (**B**) RosA, or (**C**) UrsA. Raw drug powders in PBS (pH 7.4) are shown in the bottom images; the arrows indicate the precipitated drug particles at the bottom of the vial or floating in the buffer.

After preparation, the albumin particles of curcumin, RosA, or UrsA in PBS (pH 7.4) were compared with the raw drug powder in PBS (pictures in Figure 5; A: curcumin, B: RosA, and C: UrsA). Notably, there were severe precipitations in the raw drug powders in the solution even after vigorous mixing. However, the aqueous solubility of the drug molecules increased with encapsulation in BSA owing to the amorphous form of albumin particles. Figure 5 shows the drug release kinetics of C/BSA, RosA/BSA, and UrsA/BSA formulations in the buffer at pH 7.4. While the pure drug compounds in the buffer did not give a detectable drug concentration within a period of two days in the dissolution apparatus, drug/BSA formulations provided better wettability and dispersing properties in the physiological buffer. Albumin particles containing curcumin, RosA, or UrsA showed sustained release profiles, reaching almost 100% cumulative drug release in 12 h. This suggests that sustainable ophthalmic formulations could be developed with albumin for poorly water-soluble drugs.

### 3.5. Cell Protection Activity of Drug/BSA Formulations against ROS

**Figure 6:**
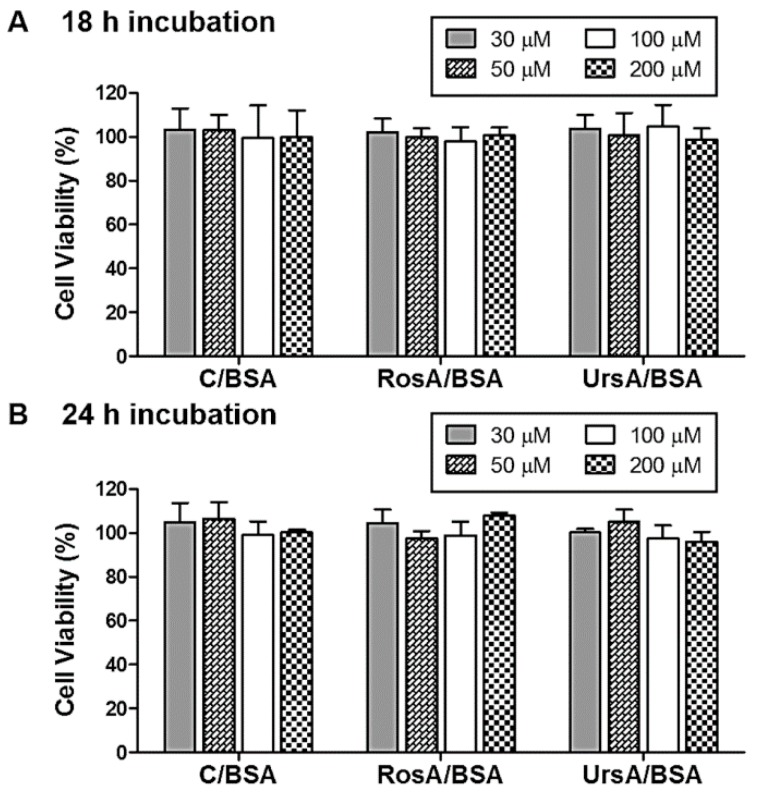
Cytotoxicity tests of adult retinal pigmented epithelial (ARPE) cells. A WST1 assay was used to evaluate cell viability percentages for the treated cells with drug/BSA particles (C/BSA, RosA-BSA, and UrsA-BSA) in various drug concentration ranges (30–200 μM). Percentages compared to the untreated control cells are shown in the bar plots for each group of cells treated with different types of drug/BSA formulations after (**A**) 18 h or (**B**) 24 h incubation.

In this study, curcumin, RosA, or UrsA/BSA formulations were tested for defense effects against oxidative stresses. Herein, hydrogen peroxide was used as an inducer for oxidative stress cascades [32,33]. Especially, in ocular physiology, retina cells have been known to be affected by reactive oxygen species generated from posterior ocular tissues (e.g., choroid and endothelium) [34]. Under normal physiologic conditions, retina cells experience endogenous oxidative stresses with higher levels of H_2_O_2_ generated in the tissues. This stress factor can propagate endogenous oxidative stress signals in the cells, resulting in aging or diseased conditions [35,36]. For this purpose, retinal pigmented epithelial (ARPE) cells were used to evaluate the cytoprotective effects of curcumin, RosA, or UrsA/BSA particles. First, we assessed any nontoxic concentration ranges of each drug formulation type using these cells. ARPE cells were incubated with the drug formulations in various drug concentrations (0, 5, 10, 30, 50, 100, or 200 µM) for 18 h or 24 h in the medium. WST-1 assay reagents were added to quantify the cell viability (%) of drug-treated cells compared to the negative cells with only the medium. Even with high concentrations after 18 h or 24 h of incubation, we observed very low cytotoxicity for curcumin, RosA, or UrsA/BSA formulations in the ARPE cells (Figure 6).

**Figure 7:**
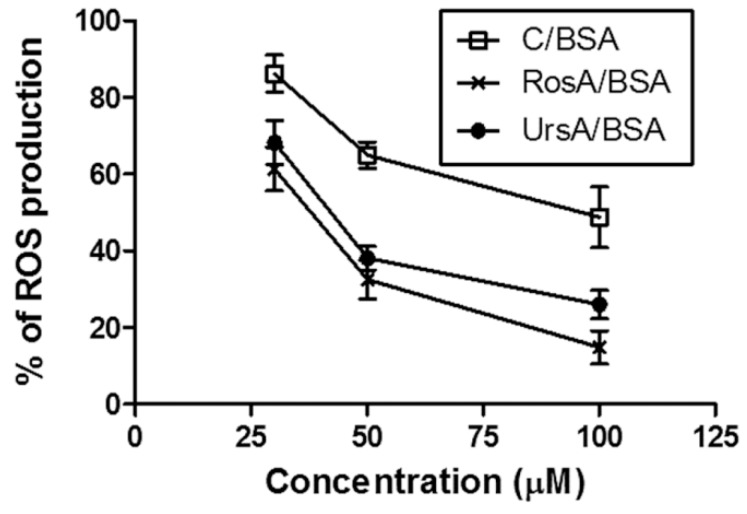
Results of the reactive oxygen species (ROS) production assay in the adult retinal pigmented epithelial (ARPE) cells. ROS production percentages were calculated by normalizing the fluorescent signals of treatment group cells (treated with 30–100 μM C/BSA, RosA/BSA, or UrsA/BSA) by the signals of control group cells (without treatment).

By incubating the normal ARPE cells with 30, 50, or 100 µM of the drug formulations for 18 h, any changes of ROS (reactive oxygen species) production levels in the cells were verified, as shown in Figure 7. Production levels of intracellular ROS were evaluated by changes in the fluorescence intensity of DCF (2′,7′-dichlorofluorescein), which are normally converted from DCFDA (2′,7′-dichlorofluorescein diacetate) in the presence of ROS [37]. At between 30 and 50 µM of drug concentrations in the BSA formulations, ROS production levels decreased faster compared with those of the drug concentrations between 50 and 100 µM. Both RosA/BSA and UrsA/BSA showed better protection from ROS production than curcumin/BSA at 50 µM.

### 3.6. Antioxidant Effects of Drug/BSA Formulations in Eye Tissues

**Figure 8:**
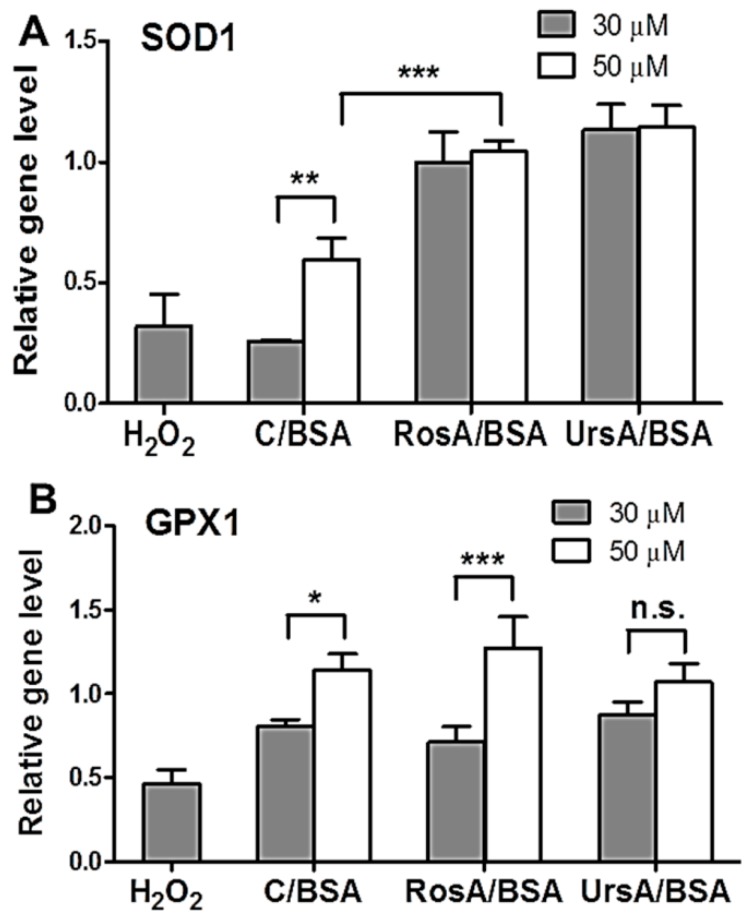
Antioxidant gene expression level analysis by qRT-PCR assay. The experiments were performed to confirm the antioxidant gene levels, such as (**A**) SOD1 and (**B**) GPX1, in the ARPE cells after treatment with formulations (up to 50 μM of drugs) and then H_2_O_2_. Relative gene levels in the treated cells were calculated by comparison with the basic gene levels in the untreated control cells. The star (*) symbol indicates the significant difference (* *p* < 0.05, ** *p* < 0.01, and *** *p* < 0.001) by one-way ANOVA test with a significance level of 0.05. n.s. means “not significant”.

Based on the ROS production experiments, the ARPE cells showed lower ROS production levels when treated with RosA/BSA or UrsA/BSA formulations than with curcumin/BSA. Furthermore, we verified gene and protein expression levels of the antioxidant enzymes in ARPE cells after treatment with the drug formulations and then with the exogenous oxidative stress factor, H_2_O_2_. Maintaining high expression levels of the protective marker genes (*SOD1* and *GPX1*) could be beneficial for protecting the tissues from oxidative stresses [8]. These gene levels, relative to the untreated control cells, were analyzed for the cells treated with curcumin/BSA, RosA/BSA, or UrsA/BSA formulations at 30 or 50 µM for 18 h and then additionally treated with H_2_O_2_. The qRT-PCR results (Figure 8) demonstrated the antioxidative, preventive efficacy of drug formulations by inducing gene expressions of *SOD1* or *GPX1* in ARPE cells. In ARPE cells, RosA/BSA and UrsA/BSA at 30 or 50 µM showed higher gene levels of *SOD1* than curcumin/BSA at these two concentrations (one-way ANOVA with *p* < 0.05). The cells incubated with curcumin/BSA at 30 M did not show a statistically significant difference in the *SOD1* gene level from those treated with H_2_O_2_ only. RosA/BSA at 50 µM gave higher *GPX1* gene levels than other formulations at 50 µM, but the difference was not statistically significant (*p* > 0.05). Curcumin/BSA and RosA/BSA showed drug concentration-dependency for inducing *GPX1* genes in ARPE cells.

**Figure 9:**
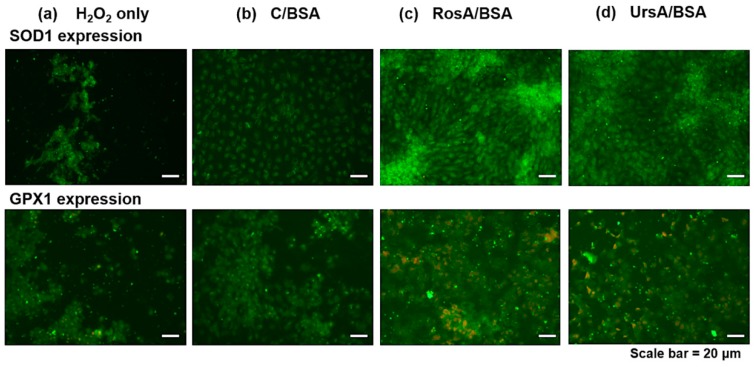
The results of immunofluorescent imaging examination of SOD1 and GPX1 protein expression in the ARPE cells. First, the cells were incubated with drug formulations (**b**: C/BSA, **c**: RosA/BSA, and **d**: UrsA/BSA) at 50 μM and then H_2_O_2_. The cells were incubated with antibodies to detect each antioxidant protein type. The images of cells treated with (**a**) H_2_O_2_ only are also displayed with the scale bar 20 μm.

**Figure 10:**
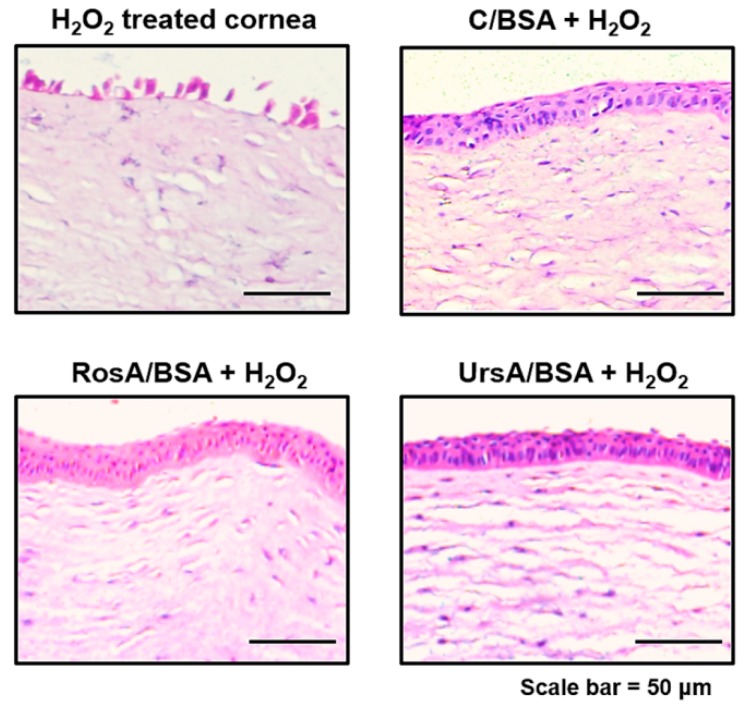
Histological examinations of rabbit cornea tissues after drug treatment (C/BSA, RosA/BSA, or UrsA/BSA formulation of 50 μM) and then H_2_O_2_ solution treatment. The cornea tissues were incubated with drug formulations for 18 h and then with 30 μg/mL of H_2_O_2_ for 4 h. Tissue sections were subjected to H&E staining and examined with a brightfield microscope (20× objective).

**Figure 11:**
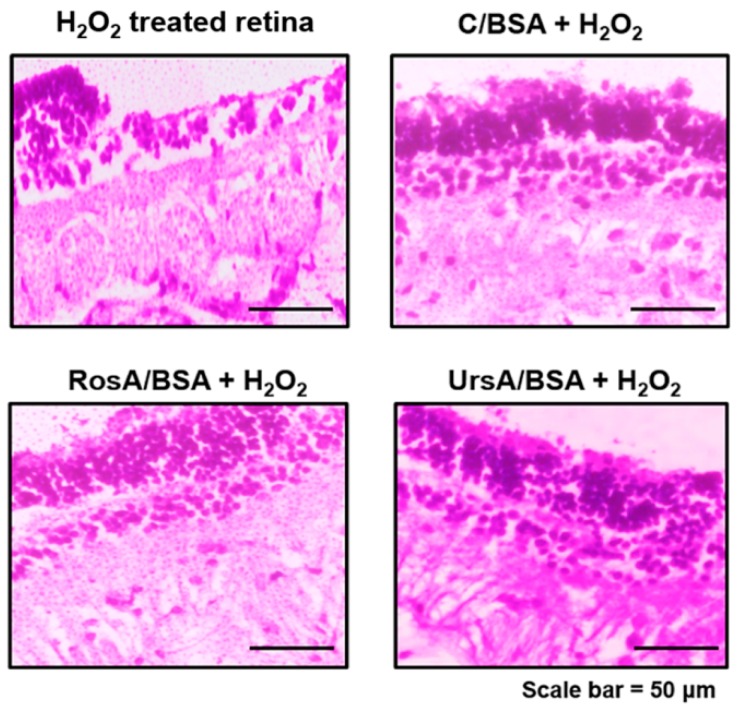
Histological examination of rabbit retina tissues after drug treatment (C/BSA, RosA/BSA, or UrsA/BSA formulation of 50 μM) and then H_2_O_2_ solution treatment. After the retinas were incubated with drug formulations for 18 h, the tissues were treated with 30 μg/mL H_2_O_2_ additionally. Tissue sections were subjected to H&E staining and examined with a brightfield microscope (20× objective).

Antioxidant enzyme expression levels in proteins were additionally verified by immunofluorescent imaging, as shown in Figure 9. After being treated with RosA/BSA or UrsA/BSA and then H_2_O_2_, the ARPE cells showed higher fluorescent intensities with higher viable cell numbers on plates than treatment with curcumin/BSA and H_2_O_2._ We also performed ex vivo studies with rabbit corneas and retinas to evaluate any protective effects of each type of drug formulation towards oxidative stress conditions resulting from H_2_O_2_. Imaging examinations were performed for H&E stained sections of rabbit corneas and retinas after H_2_O_2_ treatment only or for drug formulations and then H_2_O_2_ (Figure 10 and Figure 11). In these ocular tissues, H_2_O_2_ showed toxic effects with damaged epithelia. Overall, in both tissue types, curcumin/BSA, RosA/BSA, and UrsA/BSA showed reasonable protective effects with no signs of tissue damage even after additional treatment of H_2_O_2_, suggesting antioxidant efficacy of these drug formulations.

## 4. Conclusions

In this study, albumin particles prepared by the desolvation method increased the aqueous solubility and drug dissolution profiles of the lipophilic drugs such as curcumin, rosmarinic acid (RosA), or ursolic acid (UrsA). This is the first trial applying albumin particles encapsulating RosA or UrsA as ocular antioxidant agents. Based on the cellular studies, compared with curcumin/BSA, the colorless RosA/BSA and UrsA/BSA formulations provided better efficacy in protecting retinal epithelial cells from oxidative stresses by hydrogen peroxide. In conclusion, soluble albumin formulations of RosA or UrsA increased the expression of antioxidant enzymes at both the gene and protein levels in retina epithelial cells, compared to that of the untreated control cells, activating cellular defenses upon external oxidative stresses. Because of the antioxidant effects, RosA- or UrsA-containing albumin formulations can help to treat various ocular diseases caused by the accumulation of reactive oxygen species in the retinal and corneal tissues. With increased drug solubility and sustainable drug release, albumin formulations could provide a useful system to deliver various cytoprotectants to the eye anterior or posterior regions to prevent ocular diseases.

## Figures and Tables

**Table 1:** Particle size and zeta potential measurements of bovine serum albumin (BSA) particle formulations containing curcumin, rosmarinic acid (RosA), or ursolic acid.

Formulation		C/BSA	RosA/BSA	UrsA/BSA
**Zeta potential (mV)**				
Solution in water	Day 1	−17.04 (±1.5)	−20.69 (±1.8)	−11.66 (±1.1)
	Day 60	−18.27 (±4.3)	−26.54 (±1.3)	−12.02 (±0.6)
Freeze-dried powder	Day 1	−16.71 (±0.4)	−23.90 (±3.6)	−9.06 (±0.6)
	Day 60	−16.34 (±2.9)	−22.67 (±4.0)	−13.22 (±1.4)
**Size (nm)**				
Solution in water	Day 1	203.2 (±15.4)	156.2 (±8.3)	234.7 (±13.1)
	(PDI)	0.220 (±0.03)	0.128 (±0.06)	0.157 (±0.06)
	Day 60	354.3 (±22.3)	169.0 (±9.8)	367.0 (±14.4)
	(PDI)	0.247 (±0.05)	0.184 (±0.05)	0.151 (±0.13)
Freeze-dried powder	Day 1	257.0 (±15.4)	141.7 (±2.3)	278.2 (±9.2)
	(PDI)	0.246 (±0.03)	0.255 (±0.01)	0.196 (±0.05)
	Day 60	212.0 (±14.1)	148.7 (±1.4)	288.2 (±4.6)
	(PDI)	0.305 (±0.03)	0.090 (±0.04)	0.139 (±0.05)

Triplicated measurements of sizes and zeta potentials were performed in water. Mean values are shown with the standard deviations in parenthesis (*n* = 3). PDI = polydispersity.
